# Reduced Time CT Perfusion Acquisitions Are Sufficient to Measure the Permeability Surface Area Product with a Deconvolution Method

**DOI:** 10.1155/2014/573268

**Published:** 2014-08-12

**Authors:** Francesco Giuseppe Mazzei, Luca Volterrani, Susanna Guerrini, Nevada Cioffi Squitieri, Eleonora Sani, Gloria Bettini, Chiara Pozzessere, Maria Antonietta Mazzei

**Affiliations:** ^1^Department of Diagnostic Imaging, Azienda Ospedaliera Universitaria Senese, Viale Bracci 10, 53100 Siena, Italy; ^2^Department of Medical, Surgical and Neuro Sciences, Diagnostic Imaging, University of Siena, Viale Bracci 10, 53100 Siena, Italy; ^3^Department of Physics, Osservatorio Astrofisico di Arcetri, Largo Enrico Fermi 5, 50125 Florence, Italy

## Abstract

*Objective*. To reduce the radiation dose, reduced time CT perfusion (CTp) acquisitions are tested to measure permeability surface (PS) with a deconvolution method. *Methods and Materials*. PS was calculated with repeated measurements (*n* = 305) while truncating the time density curve (TDC) at different time values in 14 CTp studies using CTp 4D software (GE Healthcare, Milwaukee, WI, US). The median acquisition time of CTp studies was 59.35 sec (range 49–92 seconds). To verify the accuracy of the deconvolution algorithm, a variation of the truncated PS within the error measurements was searched, that is, within 3 standard deviations from the mean nominal error provided by the software. The test was also performed for all the remaining CTp parameters measured. *Results*. PS maximum variability happened within 25 seconds. The PS became constant after 40 seconds for the majority of the active tumors (10/11), while for necrotic tissues it was consistent within 1% after 50 seconds. A consistent result lasted for all the observed CTp parameters, as expected from their analytical dependance. *Conclusion*. 40-second acquisition time could be an optimal compromise to obtain an accurate measurement of the PS and a reasonable dose exposure with a deconvolution method.

## 1. Introduction

CT perfusion (CTp) is a form of functional imaging, the use of which has increased in the past few years, thanks to the diffusion of commercial software packages that allow the analysis of dynamic data sets [1–3].

**Figure 1:**
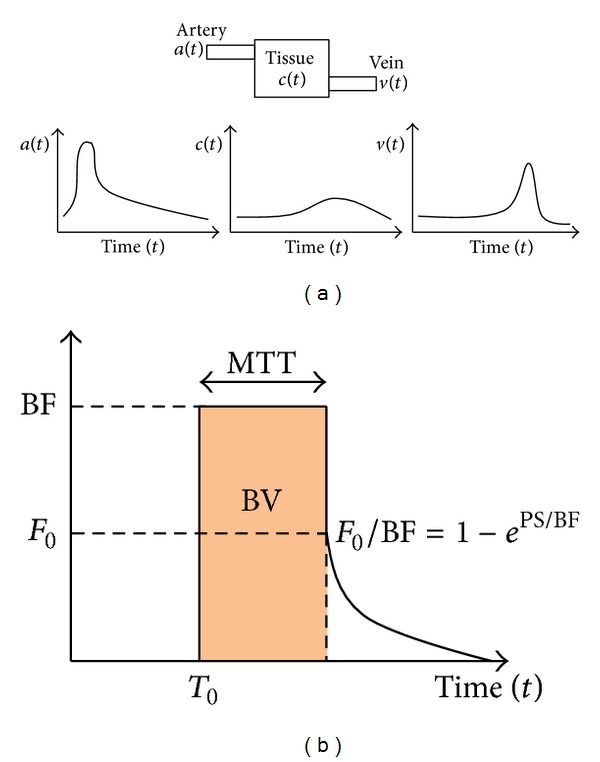
(a) Schematic representation of the time-density curves (TDCs), after the injection of a bolus of contrast material, in the three compartments (artery, tissue or compartment, and vein): *a*(*t*), *c*(*t*), and *v*(*t*). The arterial *a*(*t*) and tissue *c*(*t*) density curves are related to each other through a convolution (see (2)) of the artery curve with the impulse response function (IRF) shown in (b). (b) Scheme of the residual impulse response function (IRF) and perfusion parameters obtained through the deconvolution method (GE Medical Systems. User Guide. Milwaukee, WI: GE Medical Systems, 2002; 240-255). A measure of the IRF can be obtained by deconvolving the density curve *a*(*t*) from *c*(*t*) measured with the CTp. The CTp parameter values thus fully characterised the IRF shape as shown. In more detail, the PS is related to the diffusion coefficient of the contrast agent through the pores of the capillary endothelium into the interstitial space. In the tissue IRF, the contrast agent diffusion is related to the extraction fraction *F*_0_/BF (or the fraction of contrast agent), which remains in the intravascular space after the initial IRF response and which then diffuses exponentially into the interstitial space. The extraction fraction is thus related to the PS in the following way: *F*_0_/BF = 1 − exp⁡(PS/BF), where the parameter *F*_0_ is the blood flux measured after one mean transit time, MTT (i.e., when the contrast bolus is passed).

CTp requires dynamic contrast material-enhanced imaging involving intravenous injection of a contrast material bolus and sequential imaging to simultaneously monitor changes in the iodinated tracer concentration as a function of time both in the tissue of interest and in a vessel that is used as an input function to determine perfusion parameters of a given tissue, such as the blood flow (BF), the blood volume (BV), the mean transit time (MTT), and the capillary permeability surface (PS). The latter is considered a functional CT surrogate marker of tumoural angiogenesis and in this sense it can be used as an aid to carefully evaluate the response to therapy in oncologic patients, especially with the new therapies [4]. Calculation of these parameters is strictly dependent on the arterial time-density curves (TDCs) obtained by positioning a region of interest (ROI) on the input function vessel [5]. Software packages for CTp analysis typically extract the TDC and subsequently derive the perfusion parameters of given tissue by using computational models, such as a graphic analysis of a two-compartment model, “the so-called Patlak plot,” or a deconvolution technique based on the time invariant linear compartmental model, which liken the relationship between the arterial, tissue, and possibly the venous enhancement, to a mixing compartment (Figure 1) [6, 7]. The Patlak plot quantifies the PS parameter by relating the amount of tracer accumulated in the tissue (*C*(*t*)) to its concentration in the blood (*a*(*t*)):

(1) $$\begin{array}{c} c\left(t\right)=\text{BV}a\left(t\right)+\text{PS}\overset{t}{\underset{0}{\int }}a\left(t^{\prime }\right)dt^{\prime }, \end{array}$$

where the BV and PS are free parameters (intercept and slope, resp.) of a linear regression for *c*(*t*)/*a*(*t*) against ∫_0_^*t*^ *a*(*t*′)/*dt*′/*a*(*t*). The deconvolution model (DM) computes the residual impulse response function (IRF) that relates the arterial and tissue TDCs:

(2) $$\begin{array}{c} c\left(t\right)=\left(a⊗\text{IRF}\right)\left(t\right). \end{array}$$

**Figure 2:**
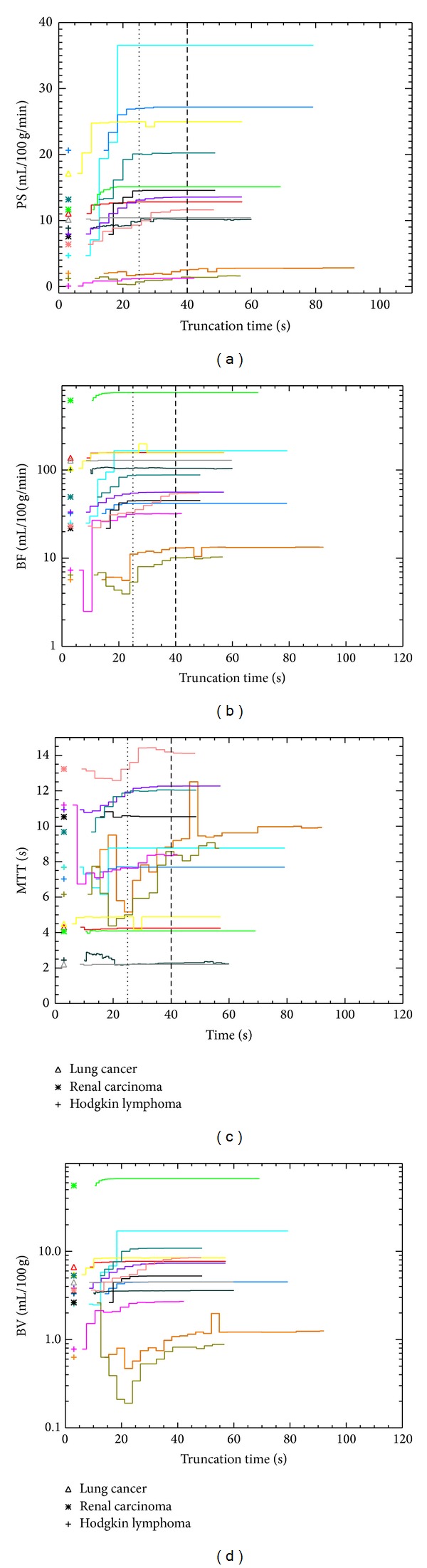
The trend of all the perfusion parameters measured using CT Perfusion 4D platform against the truncation time for the 14 CTp studies: the dotted vertical lines at 25 seconds and 40 seconds identify the three temporal intervals where the data are collected; variation of the truncated PS (a), BF (b), MTT (c), and BV (d) measurements.

Once *a*(*t*) and the IRF are deconvolved the perfusion parameters are defined as in Figure 2. The PS is related to the diffusion coefficient of the contrast agent through the capillary endothelium into the interstitial space. In the tissue IRF, the contrast agent diffusion is related to the extraction fraction *F*_0_/BF, or the fraction of contrast agent, which remains in the intravascular space after the initial IRF response and which then diffuses exponentially into the interstitial space. The extraction fraction is thus related to the PS according to Figure 1(b) and (3):

(3) $$\begin{array}{c} \frac{F_{0}}{\text{BF}}=1-e^{\text{PS}/\text{BF}}, \end{array}$$

where the parameter *F*_0_ is the blood flow measured after one mean transit time (i.e., when the contrast bolus is passed), *F*_0_ = IRF(*T*_0_ + MTT).

In both methods (1) and (2) the PS is therefore a derived quantity that requires first measuring other primary parameters. With the Patlak plot it is necessary to first measure and model the arterial and tissue TDCs and finally to perform a linear regression [8]. In order to obtain an accurate linear regression, and thus PS, it is necessary that the contrast not leave the tissue during the measurements and long radiation exposures for sufficient data for fitting [8]. The DM does not require to model *a*(*t*) and *c*(*t*) but only to measure their temporal trend and the tissue perfusion parameters are directly computed as a function of the height of the BF [9]. Beyond this computational advantage, the DM should be able to measure the PS with a short duration acquisition, avoiding unnecessary dose exposure to the patient [10]. In particular the method for obtaining PS involves separating contribution of the delivery of contrast medium via the supply artery from the enhancement of the extracellular compartment of the tissue of interest by performing a deconvolution of the tissue TDC with respect to that of the supply artery. It is common practice to acquire TDCs over a period of a minute or more, in part based on a reasoning that the additional data in a time-series that covers a longer interval should provide a more stable and accurate estimate of PS. During the first passage of contrast media, the large concentration gradient between blood and tissue maximises the attenuation changes mediated by a relative PS. As the blood and tissue concentrations gradually approach equilibration, the PS becomes irrelevant, and later, as excretion further lowers the blood concentration, a small reverse (tissue to blood gradient) will lead to clearance of the contrast medium from the tissue, but with little change seen between the individual timepoints. Thus, the question exists as to whether time-points beyond the first passage of contrast media contribute significantly to producing an accurate estimate of PS. Furthermore it would clearly be convenient to apply the shortest acquisition durations, anyhow without compromising the CTp parameter measurements. However the deconvolution algorithm, and thus the accuracy in the CTp estimates, depends on the number of experimental points derived for *a*(*t*) and *c*(*t*) curves. In other words, a given number of points are necessary to reach the algorithm convergence, and this, in turn, is a function of both the CTp acquisition duration and its temporal resolution.

Examining the previous consideration, in the present study we investigate this hypothesis for an unbiased sample of pathologies, in order to determine the feasibility of a short duration tumour perfusion acquisition protocol that would limit the dose exposure to the patient.

## 2. Materials and Methods

### 2.1. Patients and CTp Examination

Our study had institutional review board approval and a written informed consent was obtained from all patients. To realise our work, fourteen consecutive CTp studies, conducted in 11 patients (mean age 52.5 years; range 30–75 years; 7 males and 4 females) for lung cancer during chemotherapic treatment (LC1, LC2, and LC3), Hodgkin lymphoma (HL2, HL3, HL5, and HL7 active, i.e., at staging time with a positive PET imaging, and HL1, HL4, and HL6 inactive, i.e., in complete response [CR] after therapy according to the revised Cheson criteria, inactive residual mass with a negative PET imaging), and renal cell carcinoma (RCC1, RCC2, RCC3, and RCC4), were randomly selected from a cohort of 100 CTp studies performed in our department from January 2013 to April 2013. The CTp studies were performed with a 64-section MDCT scanner (VCT, GE Healthcare, Milwaukee, WI, USA). A supervising radiologist (9 years of experience in CTp) identified the tumour and then placed the predefined scan volume (80 mm for shuttle axial technique and 40 mm for cine technique) in the *z*-axis to cover the lesion for the CTp study. Cine technique was used when the lesion was smaller than 20 mm. The dynamic cine acquisition consisted of 8 contiguous sections, collimated to 5 mm, with temporal resolution of 1 second by using a cine-mode acquisition without table movement and with the following parameters: 100 Kv, 80 mAs, rotation time 0.5 s, and scan field of view of 50 cm, whereas it consisted of 8 contiguous sections, collimated to 5 mm, with temporal resolution of 2.8 seconds by using a shuttle-mode acquisition with table movement (17 to 33 passes) and with the following parameters: 100 Kv, 80 mAs, rotation time 0.4 s, and scan field of view of 50 cm. The median acquisition time of CTp studies was 59.35 seconds (range 49–92 seconds). For the CTp study, 100 mL of Iomeprolo (Iomeron 400; Bracco, Milan, Italy) was administered intravenously at a flow rate of 5 mL/s followed by 40 mL of saline solution at the same flow rate. Scan acquisition commenced 5 to 10 seconds after the start of the contrast material injection, according to the lesion location, in order to ensure the acquisition of a little nonenhanced baseline images at the tumour level to allow the software to plot the enhancement change over time. Patients were allowed to breathe gently during the dynamic scan acquisition. In all patients, restraining bands were placed around the abdomen or thorax to limit respiratory movements. In Table 1, the main features of the 14 CTp studies selected to realise our work are summarised.

### 2.2. CTp Measurements

Image analysis was performed in consensus by 2 radiologists, who were experienced in the analysis of CTp studies (with 9 and 2 years of experience in CTp, resp.). All 14 CTp studies were analyzed by using the current version of commercial software (Body Tumour CT Perfusion software version 4D; GE Healthcare Technologies). A processing threshold between 0 and 120 Hounsfield units (HU) was utilised. The arterial input was determined by placing a circular ROI of no more than half the arterial diameter, within the aorta. A TDC for the entire acquisition time of each study was generated automatically. Tumour BF, BV, MTT, and PS were calculated with repeated measurements (*n* = 305), while truncating the TDCs at different time values in the 14 CTp studies selected. In particular perfusion parameters of the selected tissue were measured on a circular or oval ROI, larger than 70% of the minimum diameter of the tumour, which was chosen to incorporate the solid-appearing part of the target lesion. For each patient the arterial and tissue ROI as well as the start of the TDCs (taken as the last timepoint before the start of the upslope of the arterial TDC) were maintained fixed. A series of BF, BV, MTT, and PS measurements were then obtained by changing the truncation time of the TDCs, positioning the first cursor by the last preenhancement image and the second cursor at any time indicating the temporal resolution (i.e., at any 0.5 or 2.8 seconds for cine and shuttle acquisition, resp.) from that, to allow the processing of the data to the end of the TDC. In the following, we refer to the temporal position of the second cursor as the “truncation time” (Tt). This allows, for each patient, elaborating modified CTp data sets varying the duration of the dynamic phase.

### 2.3. Statistical Analysis

To verify the accuracy of the deconvolution algorithm, we investigated the variation of the truncated PS value against the truncation time. Since large relative errors affect any single measurement, we limited the analysis to a direct comparison of the PS values without considering the standard deviation provided by the software across the tumour ROI for each truncation time. Then, for each patient we have collected the truncated data in three temporal intervals, chosen in order to maximise the gap in the standard deviations between an interval and its next. The analysis was also performed for all the parameters obtained (BF, MTT, and BV) using CTp 4D platform.

To evaluate whether the CTp metrics were able to distinguish between active and inactive tumours even with relatively short scanning, we performed a Kolmogorov-Smirnov (K-S) test limited to the Hodgkin lymphoma where we have 4 active (i.e., at staging time with a positive PET imaging) and 3 inactive tissues (i.e., in complete response [CR] after therapy according to the revised Cheson criteria, inactive residual mass with a negative PET imaging) (i.e., 109 versus 52 measurements, resp.).

## 3. Results

The mean and variation in the tumour PS, BF, MTT, and BV values obtained with DM processing for different choices of truncation time (Tt) are shown in Figure 2. The CTp 4D was able to process the data in all cases. The collection of the truncated measurements in three temporal intervals (i.e., those Tt values that maximise the gap in the standard deviations between an interval and its next) yields the cuts at 25 s and 40 s for active tumours (HL2, HL3, HL5, HL7, LC1, LC2, LC3, RCC1 RCC2, RCC3, and RCC4), while for necrotic tissues (HL1, HL4, and HL6) they are 25 s and 50 s. The relative (with respect to the mean PS, in a given interval) standard deviations are listed in Table 2. A relative error of 0.00 means that the PS remained constant within the truncation interval. Regarding the analysis of standard deviation in truncated PS, a visual inspection of Figure 2 reveals that, in the majority of the cases, the PS values rise up for 25 s with a later flattening of the trend. This suggests that the algorithm needs about 25 s to have sufficient coverage of the TDCs to perform a reliable deconvolution, after which the results remain stable. Comparing the values listed in Table 2, in most of the cases (13/14), the largest variability happens within 25 s; in 45% of the patients (HL2, LC1, LC3, RCC1, and RCC3), the PS value becomes constant, truncating the data after 25 s. The same happens in 91% (10/11) of the active tumours, after 40 s, the only exception being HL5 but with a relative error smaller than 1%. Necrotic tissues (HL1, HL4, and HL6) are characterised by noisier data and required at least 50 s to obtain a standard deviation lower than 1%. This reflects the low enhancement in their images (i.e., a poorly determined TDC *c*(*t*)), which is due to limited penetration of the contrast in the necrotic tissues. Comparing the 7 HL (crosses in Figure 2), the trends of active and inactive pathologies are clearly separated, as confirmed by the K-S (test probability, 1.5 × 10^−32^).

## 4. Discussion

A major limitation of CTp studies is the significant dose of radiation to the patient, mainly due to the long exposure times (>60 s) [10–12]. Tognolini et al. have recently demonstrated that CTp using the deconvolution method does not require a scanning time of more than 20–30 s for the BF computation in a rat tumour model [13]. To our knowledge, no studies have demonstrated the same possibility in computing CTp parameters in humans. PS parameter is a derived quantity that requires one to first measure primary parameters of CTp, such as BF, BV, and MTT. With the Patlak plot a long acquisition time seems to be necessary to achieve an accurate representation of the first pass of the injected contrast material bolus or vascular phase [*a*(*t*)] and a second phase, or interstitial phase [*c*(*t*)], to depict the dynamic characteristics of lesion enhancement and to obtain an accurate linear regression and thus a reliable PS. The DM does not require one to model *a*(*t*) and *c*(*t*) but only to measure their temporal trend. Beyond this computational advantage, the DM allows one to measure the PS with a short acquisition time. In fact, it is sufficient to have enough observed points in the TDCs to stabilise the deconvolution algorithm and measure primary parameters. Once the algorithm is stable, increasing the acquisition time adds little or no information about PS or for the other parameters. In our work, ~40 seconds of acquisition time was found to be sufficient to obtain a reliable IRF deconvolution and accurate PS value in the absence of those regarding necrotic tissues (e.g., checked after chemotherapy), for which slightly longer exposures (50 seconds) yield more consistent results (relative error <1%). The main limitation of this study is the absence of a direct comparison with other computation models, as the Patlak plot, which could be the subject of a further study.

## Figures and Tables

**Table 1:** Main features of the 14 CT perfusion studies selected to realise our work.

Patient	Perfusion technique	Time of acquisition (s) (mean 59.35 s)	Temporal resolution (s)	Number of measurements
HL 1 (inactive)	Shuttle	92	2.8	22
HL 2 (active)	Shuttle	56	2.8	23
HL 3 (active)	Shuttle	72	2.8	21
HL 4 (inactive)	Shuttle	57	2.8	17
HL 5 (active)	Cine	59	0.5	48
HL 6 (inactive)	Shuttle	49	2.8	13
HL 7 (active)	Shuttle	57	2.8	17
LC 1	Shuttle	57	2.8	18
LC 2	Shuttle	57	2.8	19
LC 3	Shuttle	60	2.8	19
RCC 1	Shuttle	49	2.8	12
RCC 2	Shuttle	49	2.8	13
RCC 3	Cine	69	0.5	49
RCC 4	Shuttle	48	2.8	14

CTp studies conducted for Hodgkin lymphoma (HL, n = 7; 3 of which were after treatment), lung cancer (LC, n = 3), and renal cell carcinoma (RCC, n = 4).

**Table 2:** Relative PS error (%).

Patient	Tt > 0 s	Tt < 25 s	25 s ≤ Tt < 40 s	Tt > 40 s
HL 1	17.00	13.00	16.00	6.40 (1.3)
HL 2	29.00	65.00	0.00	0.00
HL 3	6.10	12.00	0.34	0.00
HL 4	36.00	61.00	24.00	7.70 (0.96)
HL 5	36.00	49.00	1.20	0.78
HL 6	5.40	3.10	1.60	1.10 (0.38)
HL 7	15.00	18.00	1.30	0.00
LC 1	3.30	5.20	0.00	0.00
LC 2	8.40	14.00	1.20	0.00
LC 3	18.00	1.50	0.00	0.00
RCC 1	14.00	24.00	0.00	0.00
RCC 2	14.00	20.00	0.45	0.00
RCC 3	4.30	73.00	0.00	0.00
RCC 4	19.00	15.00	6.00	0.00

For each patient (column 1), the standard deviation of PS over the tumour ROI/the truncation interval (expressed as a % of the PS mean) is given for each of the truncation intervals (column 2) and in subsequent truncation time (Tt) intervals (columns 3, 4, and 5). For necrotic lymphomas (i.e., HL1, HL4, and HL6) we also list in parenthesis the value shifting the last Tt cut at 50 sec.

HL: Hodgkin lymphoma; LC: lung cancer; RCC: renal cell carcinoma.
